# Highly Active Ice‐Nucleating Particles at the Summer North Pole

**DOI:** 10.1029/2021JD036059

**Published:** 2022-03-17

**Authors:** Grace C. E. Porter, Michael P. Adams, Ian M. Brooks, Luisa Ickes, Linn Karlsson, Caroline Leck, Matthew E. Salter, Julia Schmale, Karolina Siegel, Sebastien N. F. Sikora, Mark D. Tarn, Jutta Vüllers, Heini Wernli, Paul Zieger, Julika Zinke, Benjamin J. Murray

**Affiliations:** ^1^ School of Earth and Environment University of Leeds Leeds UK; ^2^ School of Physics and Astronomy University of Leeds Leeds UK; ^3^ Department of Space, Earth and Environment Chalmers University Gothenburg Sweden; ^4^ Bolin Centre for Climate Research Stockholm University Stockholm Sweden; ^5^ Department of Environmental Science Stockholm University Stockholm Sweden; ^6^ Department of Meteorology Stockholm University Stockholm Sweden; ^7^ School of Architecture, Civil and Environmental Engineering École Polytechnique Fédérale de Lausanne Lausanne Switzerland; ^8^ Now at Institute of Meteorology and Climate Research Karlsruhe Institute of Technology Karlsruhe Germany; ^9^ Institute for Atmospheric and Climate Science ETH Zürich Zürich Switzerland

**Keywords:** Arctic, ice‐nucleating particles, ice, mixed‐phase clouds

## Abstract

The amount of ice versus supercooled water in clouds is important for their radiative properties and role in climate feedbacks. Hence, knowledge of the concentration of ice‐nucleating particles (INPs) is needed. Generally, the concentrations of INPs are found to be very low in remote marine locations allowing cloud water to persist in a supercooled state. We had expected the concentrations of INPs at the North Pole to be very low given the distance from open ocean and terrestrial sources coupled with effective wet scavenging processes. Here we show that during summer 2018 (August and September) high concentrations of biological INPs (active at >−20°C) were sporadically present at the North Pole. In fact, INP concentrations were sometimes as high as those recorded at mid‐latitude locations strongly impacted by highly active biological INPs, in strong contrast to the Southern Ocean. Furthermore, using a balloon borne sampler we demonstrated that INP concentrations were often different at the surface versus higher in the boundary layer where clouds form. Back trajectory analysis suggests strong sources of INPs near the Russian coast, possibly associated with wind‐driven sea spray production, whereas the pack ice, open leads, and the marginal ice zone were not sources of highly active INPs. These findings suggest that primary ice production, and therefore Arctic climate, is sensitive to transport from locations such as the Russian coast that are already experiencing marked climate change.

## Key Points

- The concentration of ice‐nucleating particles at the North Pole in summer 2018 was amongst the highest anywhere in the world
- Biological ice‐nucleating particles were derived from the Russian seas and perhaps associated with wind‐driven sea spray
- The concentration of ice‐nucleating particles at the surface was often different to that higher in the boundary layer where clouds form

## 1 Introduction

The Arctic climate is strongly influenced by ubiquitous low‐level mixed‐phase clouds (Kay & L'Ecuyer, 2013; Tjernstrom et al., 2012; Vüllers et al., 2021). The radiative effect of these clouds is influenced by the amount of ice and supercooled water they contain, which depends on an intricate balance of dynamical and microphysical processes (Morrison et al., 2012). Realistic representation of these processes is needed to correct model biases in the amount of supercooled liquid in mixed‐phase clouds and reduce uncertainty in feedbacks (Tan & Storelvmo, 2019).

A rare subset of the total aerosol particle population, ice‐nucleating particles (INPs), can induce primary ice production in Arctic mixed‐phase clouds when immersed in supercooled cloud droplets (Murray et al., 2012). In the summertime, Arctic marine atmospheric boundary layer temperatures are usually much warmer than those required for homogeneous freezing (≲−35°C; Herbert et al., 2015), hence heterogeneous nucleation on INPs determines the primary production of ice in clouds, at least in the absence of ice precipitating from overlying clouds (Vassel et al., 2019). Numerous INP types that can induce ice nucleation over a broad range of temperatures have been identified (Hoose & Möhler, 2012; Kanji et al., 2017; Murray et al., 2012). However, the sources and ice‐nucleating properties of INPs in the Arctic, especially the central Arctic (>80°N), are poorly defined.

INP measurements have been made around the periphery of the Arctic circle from locations close to, or on, land, but relatively few measurements have been made in the summertime central Arctic Ocean (see compilations in Welti et al. (2020) and Murray et al. (2021)). Recent research suggests that there are significant terrestrial sources of Arctic INPs including glacial dust from Svalbard (Tobo et al., 2019) and Iceland (Sanchez‐Marroquin et al., 2020), terrestrial biological aerosol from boreal forests (Schneider et al., 2021), and even particles released from thawing permafrost (Creamean et al., 2020). There are also numerous other high latitude dust sources that have not been investigated in terms of their ice‐nucleating ability (Bullard et al., 2016). Marine biogenic INPs emitted from the sea surface through bubble bursting are also thought to contribute to the INP population of the oceanic high‐latitudes (Bigg, 1996; Bigg & Leck, 2001; Hartmann et al., 2020, 2021; Ickes et al., 2020; Irish et al., 2017; Wilson et al., 2015). Sea spray is thought to produce relatively low INP concentrations, but in the absence of other sources it can dominate the INP population (McCluskey, Hill, et al., 2018; Vergara‐Temprado et al., 2017).

Ground level observations at several land‐based sites around the Arctic throughout the year showed the highest (but variable) INP concentrations during spring, summer and autumn and the lowest concentrations in winter (Wex et al., 2019). These measurements suggest that there are both marine and terrestrial INP sources around the Arctic, but it is unclear how important these sources are for clouds over the summertime central Arctic Ocean. Based on back trajectory analysis of INP measurements in the central Arctic, Bigg (1996) suggested that there is an open ocean source of INPs active at −15°C. Later, Bigg and Leck (2001) suggested the pack ice edge and bubble bursting in local leads throughout the pack ice can serve as a source of INPs. Indeed, it has been shown that there is a reservoir of INPs in the seas around the Arctic (Creamean et al., 2019; Hartmann et al., 2021; Irish et al., 2017; Wilson et al., 2015) and INP concentrations in the central Arctic decrease during the transition from Arctic summer to autumn, possibly due to the reduced availability of ice‐free marine sources (Bigg & Leck, 2001).

While it is clear that there are strong sources of INPs in the lower Arctic environment (≲80°N), it is not clear if these INPs are transported to the central Arctic. The prevailing view is that aerosol within the summertime high Arctic boundary layer experiences little effect from long‐range transport (Kupiszewski et al., 2013), and with few sources of primary aerosol in the central Arctic Ocean, sources such as local leads may be important (Bigg & Leck, 2001). However, it has also been suggested that aerosol particles can be transported from lower latitudes into the central Arctic boundary layer either through boundary layer transport or within the free troposphere with subsequent entrainment down into the boundary layer (Igel et al., 2017; Morrison et al., 2012; Schmale et al., 2021).

**Figure 1:**
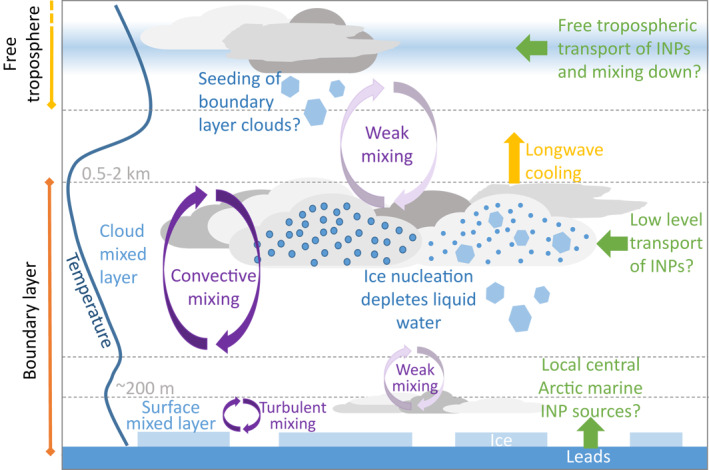
Central Arctic boundary layer structure and potential sources of INPs. The lowest part of the boundary layer (surface mixed layer) is often decoupled from the rest of the boundary layer (Brooks et al., 2017). In this paper we report INP measurements both in the surface mixed layer and in the cloud mixed layer and use these measurements to infer information about the dominant sources of INPs in the central Arctic boundary layer.

The structure of the Arctic summertime boundary layer is complex (Figure 1). The boundary layer is typically several hundred meters to over a kilometer deep, but often consists of two distinct layers: the surface mixed layer and the cloud mixed layer. These two layers are each well mixed, but often separated by a decoupling layer at ∼100–300 m that prevents efficient transport between them (Brooks et al., 2017; Shupe et al., 2013; see Figure 1). Hence, measurements at the surface are not necessarily representative of those in the cloud mixed layer. In Figure 1, we illustrate two Arctic mixed phase cloud types, one in the surface and one in the cloud mixed layer, and illustrate the three pathways we hypothesise that INP may arrive in the central Arctic in green.

In this study, we present measurements of INP concentrations close to the North Pole both in and above the surface mixed layer. The measurements were made during the Microbiology‐Ocean‐Cloud‐Coupling in the High Arctic (MOCCHA) campaign, which took place throughout August and September 2018 on the Swedish icebreaker Oden. Measurements took place while Oden was on route to the North Pole as well as when it was moored to an ice floe in the inner pack ice and drifting passively between 88 and 90°N. Samples were collected for INP analysis at both ship level (in the surface mixed layer) and using a balloon‐borne sampler in the cloud mixed layer. We use backward trajectories alongside other measurements to suggest that the source of the most active INPs reaching the North Pole is outside of the pack ice, and near the Arctic coast of Russia.

## 2 Methods

To determine the INP concentration spectra relevant for mixed‐phase clouds in the central Arctic, 48 days of sampling were conducted aboard the Swedish icebreaker Oden during Arctic summertime and the early freeze‐up period (August and September). Filter samples were collected and analyzed during the journey toward the North Pole from Svalbard whilst ice breaking, and whilst moored to an ice floe.

### 2.1 Aerosol Sampling From the Ship and the Balloon Borne Platform

For the ship‐based aerosol sampling, filters (0.4 μm pore size, polycarbonate, Nuclepore Track‐Etched Membrane Filters, Whatman) were used to collect aerosol by subsampling from a heated whole‐air inlet at a flow rate of 9 L min^−1^ (standard temperature and pressure). The inlet was mounted on the 4th deck of the ship, 25 m above mean sea level. This type of filter has been used previously for INP sampling and has a low background INP count and high particle recovery rates (Adams et al., 2020; O'Sullivan et al., 2018; Sanchez‐Marroquin et al., 2021). In addition, these filters collect aerosol across the full atmospheric size distribution with high efficiency, despite having pores of 0.4 μm (smaller aerosol particles are efficiently captured on the filter surface through diffusional processes; Adams et al., 2020).

Aerosol samples from a balloon‐borne sampler, the selective‐height aerosol research kit (SHARK; Porter et al., 2020), were collected above the surface mixed layer. All inlets were covered until sampling was started via a radio signal from the ground. Two cascade impactors were used to sample aerosol. Impactor 1 sampled at 9 L min^−1^ (Sioutas, SKC Ltd., UK) and collected on four stages (0.25–0.5 μm, 0.5–1.0 μm, 1.0–2.5 μm and 2.5–∼10 μm). For substrates on which aerosol were collected we used 25 mm diameter filters of pore size 0.05 μm (Nuclepore track‐etched membrane polycarbonate filters, Whatman, UK). In addition, we used an after filter that sits after the impactor plate to collect particles smaller than 0.25 μm (47 mm diameter Nuclepore track‐etched membrane polycarbonate filter with a pore size of 5 μm). Impactor 2 had a flow rate of 100 L min^−^^1^ (MSP Model 128, TSI, USA) and consisted of three stages (1.0–2.5 μm, 2.5–10 μm and >10 μm). In impactor 2 we used 75 mm diameter Nuclepore track‐etched membrane polycarbonate filters of pore size 0.05 μm. INP spectra from the two overlapping stages were combined in the analysis. Also, the total INP spectrum was defined by summing across all size categories (see Porter et al. (2020)). A radiosonde (S1H2‐R, Windsond, Sweden) was integrated into the instrument package to measure the temperature, pressure and relative humidity (response time of 6 s); this data could be viewed in real time from the surface. In order to choose an appropriate altitude for sampling, the radiosonde was constantly operating to provide information to the user on the ground about the SHARK altitude, boundary‐layer temperature and humidity structure as the SHARK was ascending. The top of the surface mixed layer was determined from the change in gradient of temperature with altitude. In addition, sampling was paused if the relative humidity increased above 80%, and was stopped completely before the SHARK was brought back down.

### 2.2 INP Analysis

Filters were analyzed for INP content as soon as possible after sampling, usually within 1–12 hr of being removed from the inlet. The filter samples were not frozen before offline INP analysis, due to concerns that this may affect the INP activity. Instead, they were stored at +4°C. Performing the analysis on ship soon after sampling minimized the risk of changes in the INPs on the filter since storage at any temperature is expected to affect the activity of the samples (Beall et al., 2020). The aerosol particles on the filters were washed into either 5 or 10 mL of ultra‐pure water (Millipore Alpha‐Q, with a resistivity of 18 MΩ cm at 25°C) to suspend the collected aerosol particles. These particle suspensions were then pipetted to form an array of ∼40 1 μL droplets on a hydrophobic 25 mm diameter glass slide (Hampton Research) positioned on top of a cold stage, the Microliter Nucleation by Immersed Particle Instrument, μL‐NIPI (Whale et al., 2015). The μL‐NIPI is a standard INP measurement instrument that has been benchmarked alongside a range of other INP instruments during a number of intercomparison studies (DeMott et al., 2018). The cold stage cooled at a controlled rate of 1°C min−1 until all droplets had frozen, and the freezing events were recorded in order to determine the concentration of INPs with respect to the volume of air that had been sampled through the inlet. Heat sensitivity of the collected INP samples was determined by heat treatment, where subsamples of the particle suspensions in sealed 50 mL conical centrifuge tubes were immersed in a water bath at 100°C for 30 min, before being reanalyzed using the μL‐NIPI (Daily et al., 2021).

The INP concentration data presented here is shown with the contribution from the background accounted for (i.e., the background was subtracted). The background influence on the INP concentration was determined by collating the differential nucleus concentrations for water and handling blanks, and subtracting this from the sample differential nucleus concentrations. The differential concentrations were then summed to produce the cumulative INP spectra (Sanchez‐Marroquin et al., 2021).

### 2.3 Other Measurements at Ship Level

To evaluate the concentration of dimethyl sulfide (DMS), filter samples of DMS were collected and analyzed onboard. Equivalent black carbon (eBC) concentrations were obtained from a multi‐angle absorption photometer (MAAP, Model 5012, Thermo Fisher Scientific Inc.). Particle size distribution measurements were made continuously using an aerosol spectrometer (WELAS 2300HP, Palas GmbH) for particles of size 0.15–9.65 μm, and a differential mobility particle sizer (DMPS) with a custom‐built medium Vienna‐type differential mobility analyzer (DMA) with a mixing condensation particle counter (MCPC, Model 1720, Brechtel Manufacturing Inc.) for particles of size 10–921 nm.

An ion chromatography system (ICS‐2000, Thermo Fischer Scientific, previously Dionex) was used to determine the chemical composition of the samples. Using certain standards, the concentration of chloride, nitrate, sulfate, mesylate, methane sulfonic acid, sodium, ammonium, potassium, magnesium and calcium in the sample were determined from the ion chromatograms. A synthetic sample (QC Rainwater Standard, Inorganic Ventures, USA) was used to estimate the uncertainty, which was up to 3%. More details on the method can be found in Leck and Svensson (2015).

### 2.4 Prevention of Ship Stack Pollution

There is contradictory evidence that combustion products in a ship's exhaust can influence INP populations, with some studies indicating that there may be a significant ice nucleating activity (Thomson et al., 2018), whereas others indicate that there is negligible activity (Welti et al., 2020). Therefore, rigorous sampling procedures were put in place in order to ensure that the INP concentrations measured were not affected by the ship stack emissions. The aerosol sampling inlets faced the ship's bow and the ship was maneuvered to face into the wind whenever the wind direction changed, which minimized the probability of sampling ship stack emissions. In addition, an auto‐stop for the inlet pumps was operated if aerosol concentrations increased suddenly (which would be indicative of sampling the ship stack plume), halting the sampling until aerosol concentrations returned to values similar to before the pollution event. As a precaution, the direction and speed of the wind was monitored closely, and sampling was stopped when there was a chance that the wind might introduce ship stack to the sampled aerosol. Finally, sampling was stopped if any activity that could produce aerosol was planned, including the movement of the ship, ice coring, and helicopter flights (this involved the operators being on call 24 hr a day to respond to any potential contamination). Smoking of cigarettes was also only allowed in certain areas of the ship, to ensure there was no influence on aerosol sampling. We return to a discussion of ship stack emission contamination in the results section.

### 2.5 Backward Trajectories

In order to define the potential origin of measured INPs, backward trajectories of the air reaching an altitude of 32m at the sampling location were examined. The 10‐day (only 7 days of which are used here) back trajectories were calculated using the Lagrangian analysis tool LAGRANTO (Sprenger & Wernli, 2015) with wind fields from 3‐hourly operational ECMWF analyses, interpolated to a regular grid with 0.5° horizontal resolution on the 137 model levels. The trajectory data contains the hourly positions (longitude, latitude, pressure) along the trajectory. To focus on the segments of the trajectories that can potentially be affected by surface aerosol emissions, the trajectories are only included when they were within the model boundary layer. Additionally, removal of aerosol by precipitation, which may remove the signature of upwind aerosol sources via wet deposition, has been considered by removing all the trajectory points before the precipitation event (using a threshold of 0.1 mm hr^−1^). The overall relationship with origin is unchanged by the addition of this filter, which indicates that the results were insensitive to precipitation events.

## 3 Results and Discussion

### 3.1 Ice‐Nucleating Particle Concentrations Within the Surface Mixed Layer

**Figure 2:**
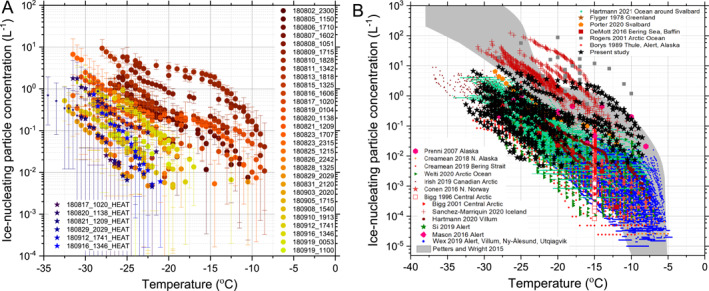
Surface mixed layer INP concentrations throughout the campaign. (a) The number of INPs per liter of air sampled was calculated using data from offline droplet freezing experiments, conducted within hours of the samples being taken. The spectra shown in blues represent samples that were heated to close to 100°C for 30 min. Background values were subtracted from the data. Sampling times varied from 6 hr to 3 days and were taken using a heated whole air inlet on the 4th deck (25 m above mean sea level) of the Oden Icebreaker. Temperature uncertainties (not shown) for the droplet freezing experiments were estimated to be ±0.4°C. The format of the key is YYMMDD_hhmm; these correspond to the start time of the filter sample in the 24 hr time format; the start and end times are in Table S1 in Supporting Information S1. (b) The data from this study are presented alongside data from the literature for ground, ship and aircraft‐based campaigns around the Arctic (Bigg, 1996; Bigg & Leck, 2001; Borys, 1989; Conen et al., 2016; Creamean et al., 2018, 2019; DeMott et al., 2016; Flyger & Heidam, 1978; Hartmann et al., 2020, 2021; Irish et al., 2019; Mason et al., 2016; Porter et al., 2020; Prenni et al., 2007; Rogers et al., 2001; Sanchez‐Marroquin et al., 2020; Si et al., 2019; Wex et al., 2019) and a compilation derived from precipitation samples (Petters & Wright, 2015).

We first present our INP concentrations derived from samples collected on the ship, which was within the surface mixed layer (Figure 2a). The concentrations of INPs measured in the surface mixed layer were highly variable, and ranged from <6 × 10^−3^ INP L^−1^ to 2 INP L^−1^ at −15°C. This resulted in INP activation temperatures ranging from −9 to −30°C for a concentration of 0.1 INP L^−1^. This is clearly contrary to what we expected in this remote location based on measurements in other remote oceanic locations around the world. For example, in the Southern Ocean, INP concentrations are systematically at the low end of what we observe here (McCluskey, Hill, et al., 2018; Murray et al., 2021; Welti et al., 2020).

The vast majority of Arctic INP measurements reported in the literature were made on land, or at least with some distance from the Pole. A summary of these measurements is given Figure 2b. These measurements clearly show that there are strong sources of INP between around 65 to 80°N (Hartmann et al., 2021; Sanchez‐Marroquin et al., 2020; Tobo et al., 2019; Wex et al., 2019). Our measurements demonstrate that the INP concentrations can also sporadically be very high in the pack ice near the North Pole. Previous measurements close to the Pole also reveal substantial variability in INP concentrations active at −15°C (Bigg, 1996; Bigg & Leck, 2001). Our results indicate sporadically higher concentrations than Bigg's results and also demonstrate that the concentration of INP can be higher than 0.1 L^−1^ at temperatures up to around −10°C. We come back to the question of where these highly active INP come from later in the paper.

Overall, it is remarkable to note that the peak concentrations measured here (2 INP L^−1^ at −15°C) are as high as the highest INP concentration reported in INP rich regions such as the mid‐latitude terrestrial environment (O'Sullivan et al., 2018; Petters & Wright, 2015). Our measurements indicate that the INP concentration spectra within the high Arctic surface‐mixed boundary layer can be extremely variable.

In order to test for the presence of proteinaceous biological ice‐nucleating material, we heated the most active sample suspensions to close to 100°C (Daily et al., 2021). Heating suspensions is known to denature the proteins responsible for ice nucleation in some biological samples, whereas mineral dust is largely unaffected. The activity of these samples was always reduced, with all of the activity above −20°C being removed (Figure 2a and Figure S1 in Supporting Information S1). Hence, it appears that the most active INPs sampled were most likely of biological origin. Atmospheric INP at lower Arctic latitudes have also been found to be heat sensitive (Hartmann et al., 2021). Together, this indicates that proteinaceous biological INP are important in the Artic.

**Figure 3:**
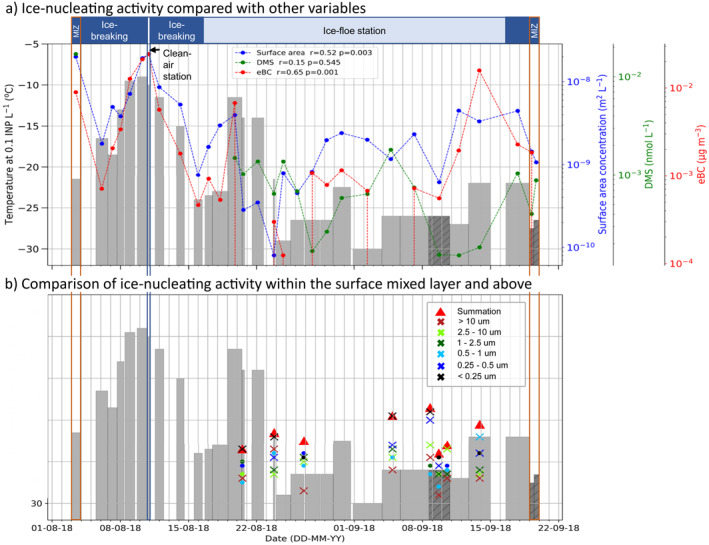
Time series showing the ice‐nucleating activity (expressed as the temperature at which a concentration of 0.1 INP L^−1^ (*T*_[INP]__= 0.1_) was observed) throughout the campaign alongside dimethyl sulfide (DMS), aerosol surface area and equivalent black carbon (eBC). In both panels a and b, the tops of the gray bars represent *T*_[INP]__= 0.1_ in the surface mixed layer (i.e., at ship level, 25 m above mean sea level), with the width of the bar representing the period over which air was sampled. The hatched gray bars are limiting values (where droplet freezing was indistinguishable from the control experiments). (a) The time series of the daily average surface area of aerosol per liter (blue), the DMS concentration (green) and the eBC concentrations (red) measured in the aerosol are shown. We also show the Pearson's r coefficient between ice‐nucleating activity and each quantity in the figure legend. (b) The values of *T*_[INP]__= 0.1_ measured above the surface mixed layer (using the SHARK balloon‐borne sampler) are shown against *T*_[INP]__= 0.1_ measured at ship level (gray bars). The red triangles are the *T*_[INP]__= 0.1_ for the summed INP concentrations across all size categories (comparable to the measurements at ship level), while the crosses indicate the *T*_[INP]__= 0.1_ associated with each size category (circles indicate limiting values). The dates for the respective periods are: MIZ 02/08/18‐03/08/18, Clean‐air station 10/08/18‐11/08/18, Ice‐breaking 03/08/18‐16/08/18, Ice floe station 16/08/18‐15/09/18, Ice‐breaking 15/09/18–19/09/18, MIZ 19/09/18.

The time series in Figure 3 shows the temperature at which a concentration of 0.1 INP L^−1^ was measured (*T*_[INP]__= 0.1_), and highlights the variability of INP concentrations at the North Pole throughout August and September of 2018. The first peak in ice‐nucleating activity was observed during a period in which the ship was breaking ice prior to being moored to an ice‐floe (i.e., prior to 16th August). It is reasonable to question whether the very high INP concentrations observed during the ice‐breaking period resulted from the ice‐breaking itself. Ice‐breaking involved frequent backward and forward motions of the ship, hence there is the potential for sampling ship emissions (such as ship stack emissions, detailed in the methodology) and aerosol resulting from ice‐breaking and the disruption of the sea surface. See the methodology for a description of the precautions taken to avoid sampling ship stack (or other) emissions. There was a pause in ice‐breaking when a clean air station was established (10th August, we sampled for 6 hr) that coincided as well with high INP concentrations. At this clean air station, the ship was moored facing into the wind, with ship‐based aerosol sources downwind of the aerosol inlets. Hence, we were confident that sampling of ship pollution and ice‐breaking aerosol were eliminated, increasing confidence that these high values in this period were indeed representative of the central Arctic Ocean. In addition, there was also a period of very high ice‐nucleating activity a few days after the ice‐floe station had been established and the ship was pointing into the wind, demonstrating that there were very active INPs that were not related to ice‐breaking or ship emissions. The time series also highlights two distinct regimes: the period up to the 23rd August was characterized by variable but often very high INP concentrations, whereas after this date INP concentrations were typically much lower. We examine the back trajectories associated with these different regimes later in the paper.

**Figure 4:**
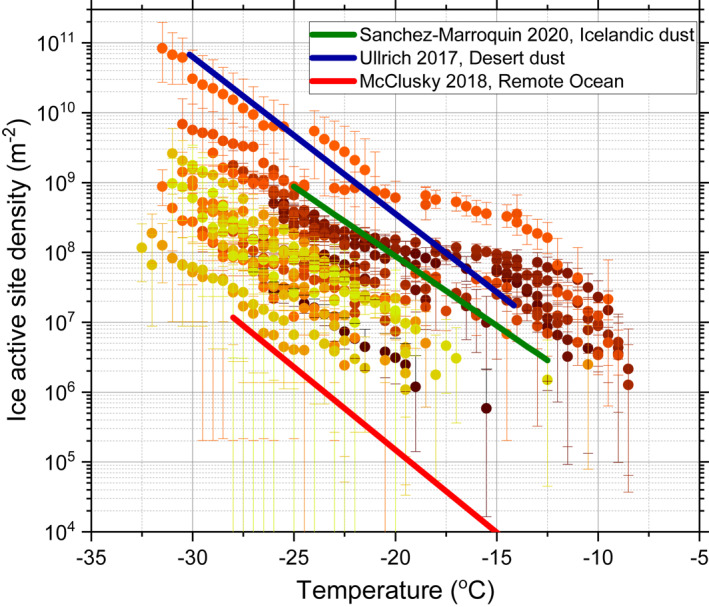
The ice‐active site density (*n*_s_) for aerosol sampled during the cruise (see Figure 2 for key to colors) compared to *n*_s_ parameterisations for desert dust (Ullrich et al., 2017), Icelandic dust (Sanchez‐Marroquin et al., 2020) and for aerosol from the North Atlantic (McCluskey, Ovadnevaite, et al., 2018), which is also consistent with values for aerosol over the Southern Ocean (McCluskey, Hill, et al., 2018).

We also show the ice‐nucleating activity in the form of ice‐active sites per unit surface area (*n*_s_) of aerosol particles in Figure 4. To calculate *n*_s_ we used the total aerosol surface area derived from the online DMPS and WELAS instruments sampling from the same whole air inlet that we used to collect the filter samples. *n*_s_ provides a means of comparing the activity of aerosol on a per unit surface area basis and we compare our results to parameterisations for marine aerosol (McCluskey, Ovadnevaite et al., 2018), desert dust (Ullrich et al., 2017), and high latitude dust from Iceland (Sanchez‐Marroquin et al., 2020). It is striking that the activity of our samples in the central Arctic are often much higher than aerosol in the north Atlantic or from over the Southern Ocean (which have a similar activity to that reported for the north Atlantic (McCluskey, Hill, et al., 2018)). It is perhaps even more striking that a subset of the samples from the central Arctic are more active than mineral dust aerosol from Iceland or low latitude deserts. This shows the aerosol at this location can be much more ice‐active than aerosol in other remote marine environments.

### 3.2 Ice‐Nucleating Particle Concentrations Above the Surface Mixed Layer

Eight flights with a balloon‐borne size‐resolved aerosol sampler were conducted while the Oden was at the ice floe station (see SI for flight details). The sampler (SHARK), was lofted to a defined height using a tethered balloon operated from the sea ice, and aerosol samples were collected into multiple size bins (see methods and Porter et al. (2020)). During these flights, we used a live link to the on‐board temperature and humidity measurements to ensure that we sampled above the surface mixed layer and thus in air decoupled from the surface, but within the boundary layer (i.e., in the cloud mixed layer). Flights occurred at 390–600 m altitude, each sampling for 3–6 hr. Sampling was paused if the RH increased above 80% to avoid sampling biases associated with hygroscopically swollen aerosol and sampling in cloud.

Given the surface mixed layer is often decoupled from the rest of the boundary layer, these measurements in principle allow us to compare INP concentrations within and above the surface mixed layer. The values of *T*_[INP]__= 0.1_ are shown in Figure 2b, and the INP spectra are shown in Figure S2 in Supporting Information S1 summed across all size bins. It should be borne in mind that, for practical reasons, the sampling durations on the ship and on the SHARK were not the same. However, it is still possible to draw conclusions from this comparison. There is evidence that there are substantial differences between the INP concentrations in the surface mixed layer compared to above it. For example, on the 5th and 8th September the *T*_[INP]__= 0.1_ (summed across all sizes) was around −18 to −19°C above the surface mixed layer, whereas it was below −26°C within it. On the 20th August, *T*_[INP]__= 0.1_ was around −23°C above the surface mixed layer, but −14°C within it. On all three days, scanning microwave radiometer and six hourly radiosonde temperature profiles confirmed that the surface mixed layer was decoupled from the rest of the boundary layer (Table S2 in Supporting Information S1). In contrast, on 13th September, the boundary layer was mainly coupled and the INP concentrations within and above the surface mixed layer were similar. However, on two other days (23rd August and 10th September) the activity in the surface mixed layer and above were similar even though it was decoupled. Overall, out of the eight SHARK samples collected above the surface mixed layer, there was one SHARK sample that had much lower ice‐nucleating activity than that in the surface mixed layer, three samples with higher activity, three with similar activity, and one that was ambiguous (due to both samples being close to the baseline of detection). This is consistent with the air at the surface sometimes being coupled to the cloud mixed layer, allowing transport of aerosol throughout the boundary layer, but at other times the measurements at the surface are not representative of those above the surface mixed layer.

The size‐resolved INP activity (*T*_[INP]__= 0.1_) is also shown in Figure 3b. In many locations around the world, supermicron aerosol dominate the INP population (Creamean et al., 2018; Gong et al., 2020; Mason et al., 2016; Porter et al., 2020; Reicher et al., 2018). However, contrary to what might be expected, the smallest size ranges of <0.25 μm contributed the most INPs on five out of the eight flights, with the 2.5–10 μm and 0.5–1 μm bins both contributing the most on one flight each. Inspection of the corresponding INP spectra associated with each bin (Figure S2 in Supporting Information S1) revealed that the particles <0.25 μm made a pronounced contribution to the INP population on the 23rd August and the 8th and 9th September. The other flights produced data mainly in the baseline for all sizes.

The coarse mode (>2.5 μm diameter) has a relatively short lifetime in the Arctic boundary layer, being removed effectively by wet scavenging processes (Leck & Svensson, 2015). Hence, it is perhaps not so surprising that the fine mode aerosol (<0.25 μm) appears to be so important in this region for the INP population. While INPs are typically thought of as being the larger particles in a size distribution (Mason et al., 2016; Porter et al., 2020), there are INPs that fall into the <0.25 μm size range that are also very active. For example, film droplet aerosol resulting from wave breaking are produced in a range of sizes centered around 100–200 nm and are often rich in organic material (Bigg & Leck, 2008; Leck & Bigg, 2005; O'Dowd et al., 2004; Orellana et al., 2011) that is thought to include small ice‐nucleating entities (Ickes et al., 2020; Schnell & Vali, 1975; Wilson et al., 2015). Ice‐nucleating macromolecules from terrestrial biological sources internally mixed with other aerosol particles might also fall into this size range (O'Sullivan et al., 2016; O'Sullivan et al., 2015; Pummer et al., 2015) and it has been proposed that fungal material, some of which is known to act as an INP (O'Sullivan et al., 2015), can fragment to form nanoparticles (Lawler et al., 2020).

### 3.3 Correlation Between INP Concentrations and Dimethyl Sulfide, Equivalent Black Carbon and Aerosol Surface Area

To investigate possible sources of the INPs we detected over the central Arctic Ocean, we correlated the ice‐nucleating activity of the aerosol with: (a) dimethyl sulfide (DMS), a product of marine biological activity, particularly in the marginal ice zone (MIZ, the transitional zone between open sea and dense pack ice; Leck & Persson, 1996); (b) eBC, based on aerosol absorption at 637 nm; and (c) aerosol surface area, derived from size distribution measurements. We present the time series for aerosol particle surface area, DMS and eBC concentrations, as well as the Pearson's r coefficient between ice‐nucleating activity and each quantity in Figure 3a.

DMS is found in the marine atmosphere, originating from the metabolites of some marine algae (Leck & Persson, 1996; Lohmann & Leck, 2005). Hence, the presence of DMS indicates that an air mass has origins in a location rich in biological activity, which may also be expected to correlate with marine biological INP sources. DMS is thought to be relatively short‐lived in the atmosphere, with a lifetime on the order of 1–3 days (Kerminen & Leck, 2001; Khan et al., 2016). Therefore, it is a useful indicator for the interaction of air masses with the MIZ at the outer edge of the pack ice region, and possibly the open leads or melt ponds within the pack ice if they were producing DMS at that time.

The concentration of DMS during the cruise was highest during the outbound 24 hr MIZ station (2nd‐3rd August), where the ship was close to open water, but was variable whilst in the pack ice (Figure 3), and remained relatively low during the inbound MIZ station (19th September). The data in Figure 3 clearly shows that there is no obvious correlation between DMS and INP activity (r = 0.15) suggesting that MIZ marine biogenic sources exerted little influence on the measured INP concentrations. This does not rule out marine sources of INP further afield.

Equivalent black carbon (eBC) is a quantity derived from aerosol absorption and is the equivalent black carbon mass concentration needed to produce the observed absorption. Other aerosol types such as dust, brown carbon or other organic aerosol might also produce absorption, thus potentially contaminating the small signal we observed. However, absorption at 637 nm by BC is much stronger than for other materials, hence the signal is most likely dominated by BC. BC is produced through a range of combustion processes, including biomass burning, wildfires and fossil fuel combustion, which all occur remote from the central Arctic. Other potential contributors to the absorption signal, such as dust or brown carbon are also remote from the central Arctic. Rigorous procedures were in place to ensure that BC (and other aerosol) from the ship stack did not affect measurements (see methods for details). Therefore, eBC is used here as an indicator of long‐range transport. The literature indicates that BC is a relatively ineffective ice nucleator under mixed‐phase cloud conditions (Adams et al., 2020; Chen et al., 2018; Schill et al., 2020; Vergara‐Temprado, Holden et al., 2018; Welti et al., 2020), hence we would not necessarily interpret a positive correlation as an indication of ice nucleation by BC. However, combustion processes are thought to be a source of ice‐nucleating aerosol, even if BC itself is not an effective INP (Barry et al., 2021; Jahn et al., 2020; Umo et al., 2015; Wagner et al., 2018). Thus, a correlation between BC and INP concentrations would indicate that aerosol particles transported along with BC from outside the area of the central Arctic Ocean nucleate ice. Wildfires around the Arctic are a potential source of BC, and we note that during the cruise there were persistent Siberian wildfires, as can be seen using the NASA Worldview satellite imagery tool (e.g., clear skies on the 14th August reveal widespread fires; https://worldview.earthdata.nasa.gov/). There is also industry, shipping and mining along the Arctic coast of Russia, and the role of gas flaring is thought to be a particularly important source of BC (Stohl et al., 2013). The overall correlation between eBC and ice‐nucleating activity (expressed as *T*_[INP]__= 0.1_) is 0.65. There are periods when the eBC concentration and *T*_[INP]__= 0.1_ track one another, but there are other periods where they vary independently. For example, between the 6th and the 19th August eBC and *T*_[INP]__= 0.1_ are highly correlated. However, on the 20th August the eBC decreases dramatically, but the INP remain elevated. Conversely, later in the campaign, the eBC values are some of the highest we measured, but the ice nucleating activity remains very low. Hence, we concluded that the eBC indicates long range transport, but this transport only sometimes coincides with remote sources of INP.

The surface area concentration of the bulk aerosol follows a similar trend to the eBC concentrations, but with a slightly weaker correlation with the *T*_[INP]__= 0.1_ (r = 0.52). Similar to the eBC, the surface area and *T*_[INP]__= 0.1_ 6th and the 19th August correlate strongly, but on the 20th August the aerosol surface area decreases by around an order of magnitude without a corresponding decrease in *T*_[INP]__= 0.1_. This indicates that the variability in INP concentrations at the North Pole is not simply driven by aerosol surface area, rather that some specific component(s) of the aerosol population are ice‐active and these particles are most likely associated with specific sources at latitudes further south than the MIZ.

### 3.4 Trajectory Analysis of Aerosol Collected in the Central Arctic

**Figure 5:**
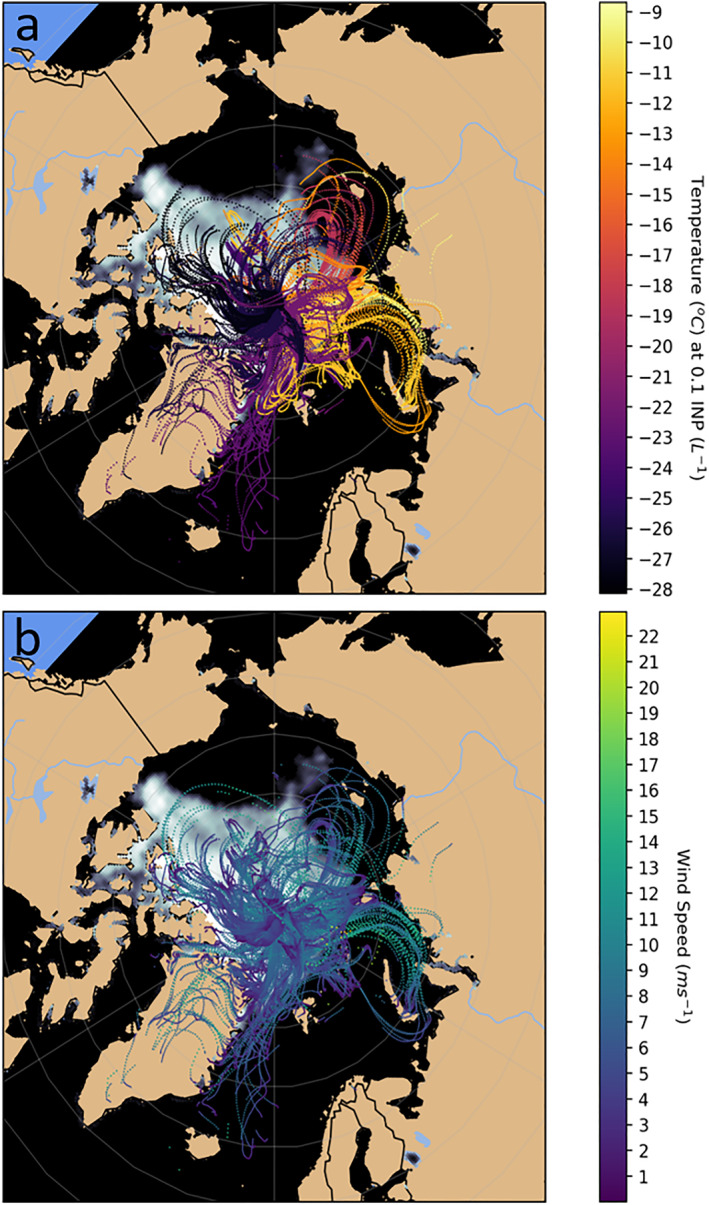
Backward trajectories over 7 days, starting at the ship location, for the INP samples taken throughout the campaign. Trajectories were launched every hour during the sampling period, and each point represents an hour in time along the back trajectory. The starting height for the trajectories was 32 m above mean sea level. Any points along the trajectories which were above the model boundary layer and any points along the trajectory that preceded precipitation events (>0.1 mm hr^−1^) were removed. Hence, any potential sources of INP in the boundary layer are neglected if they occurred prior to a precipitation event (we assume precipitation removes INPs). (a) The color of the trajectories represents the temperature at which 0.1 INP L^−1^ was measured for that sampling period. (b) The color of the trajectories represents the wind speed for each point along the trajectory. The sea ice extent is from the NASA National Snow and Ice Data Center (Maslanik & Stroeve, 1999).

Backward trajectories from the sampling location near the surface are presented in Figure 5a. We only show points that are within in the boundary layer and for a maximum of 7 days. The figure shows a clear relationship between the origin of the aerosol, considered here as the boundary layer points along the trajectories, and the measured INP concentrations. The origin of the air with the most active INPs is around the Russian Arctic coast including the Barents, Kara and Laptev Seas. Out of the 30 filter runs, those filters with the highest INP activity (the top 20% of filters; −9°C ≥ *T*_[INP]__= 0.1_ > −13.5°C) sampled air masses originating over the Barents and Kara Seas. The next seven highest (23%; −13.5°C ≥ *T*_[INP]__= 0.1_ > −22°C) filters, in terms of INP activity, sampled air originating from over the Laptev and East Siberian Seas. The next six filters with lower INP activity (20% of filters; −22°C ≥ *T*_[INP]__= 0.1_ > −25°C) sampled air that originated off the eastern coast of Greenland from over both the pack ice and open ocean. The 11 filters with the lowest INP activity (bottom 37% of filters; −25°C ≥ *T*_[INP]__= 0.1_ ≥ −30°C) all sampled air which mostly originated from the pack ice adjacent to North America (also see Figure S3 in Supporting Information S1).

It is striking that the trajectories with the lowest INP concentrations spent most of the preceding seven days over the pack ice and to some extent over the MIZ. These results indicate that, during this campaign, open leads, sea ice and the MIZ were at most weak sources of INPs. This conclusion contrasts with previous suggestions (Bigg & Leck, 2001; Hartmann et al., 2020) of a local source of biogenic particles and INP from open leads.

The highest ice‐nucleating activities from sampled aerosol originating along the Russian coast were also correlated with high wind speeds along the trajectories, that is, in the region of the aerosol origin (Figure 5b). This, together with the heat tests and INP size information, point to a wind‐driven marine biological source of INPs associated with organic‐rich film droplet sea spray aerosol. There were trajectories with high wind speeds over the North American continent, the pack ice and the coast of Greenland, but the ice‐nucleating activity for these was not greatly enhanced. Hence, our results are consistent with a strong source of highly active INPs in the coastal marine waters of northern Russia that were aerosolized during windy conditions. Marine waters elsewhere in the world are thought to produce aerosol with relatively low ice nucleating activities (Vergara‐Temprado et al., 2017; see Figure 4); however, our results suggest that the shallow seas off the Russian coast might be relatively strong sources of highly active INPs. Composition analysis of the aerosol during the peak ice nucleation activity on the 11th August and the 19th August is consistent with a marine source for many of these aerosol particles (samples were rich in Na, Cl and sulfate; see Table S3 in Supporting Information S1). Hence, the question is why the aerosol from near the coast of Russia is so much more active than aerosol derived from other marine locations such as the North Atlantic and the Southern Ocean (McCluskey, Hill, et al., 2018; McCluskey, Ovadnevaite, 2018).

A major difference to the North Atlantic and Southern Ocean is that the shallow seas off the coast of northern Russia are strongly influenced by riverine input from Russia that is rich in organic material, silt and nutrients (Ahmed et al., 2020; Juhls et al., 2019). In fact, much of the dissolved organic matter in the Arctic Ocean is derived from river input (Juhls et al., 2019) and the discharge of these rivers (and the amount of dissolved organic carbon flowing into the sea via rivers) is increasing (Ahmed et al., 2020; Juhls et al., 2020). It has been shown that melting permafrost, which is known to enter river water (Juhls et al., 2020), harbors copious quantities of warm temperature INPs (Creamean et al., 2020). Also, it has been shown that river water elsewhere in the world can contain highly active INPs (Knackstedt et al., 2018; Larsen et al., 2017). Hence, it is possible that the highest INP concentrations we detected at the North Pole were derived from marine waters rich in terrestrially derived ancient biological INPs. Alternatively, marine biology fertilised by the nutrient rich waters on the continental shelf may produce more INPs than are present in remote marine locations. A measurement campaign to quantify the INP content of the waters off the coast of Russia is clearly required.

**Figure 6:**
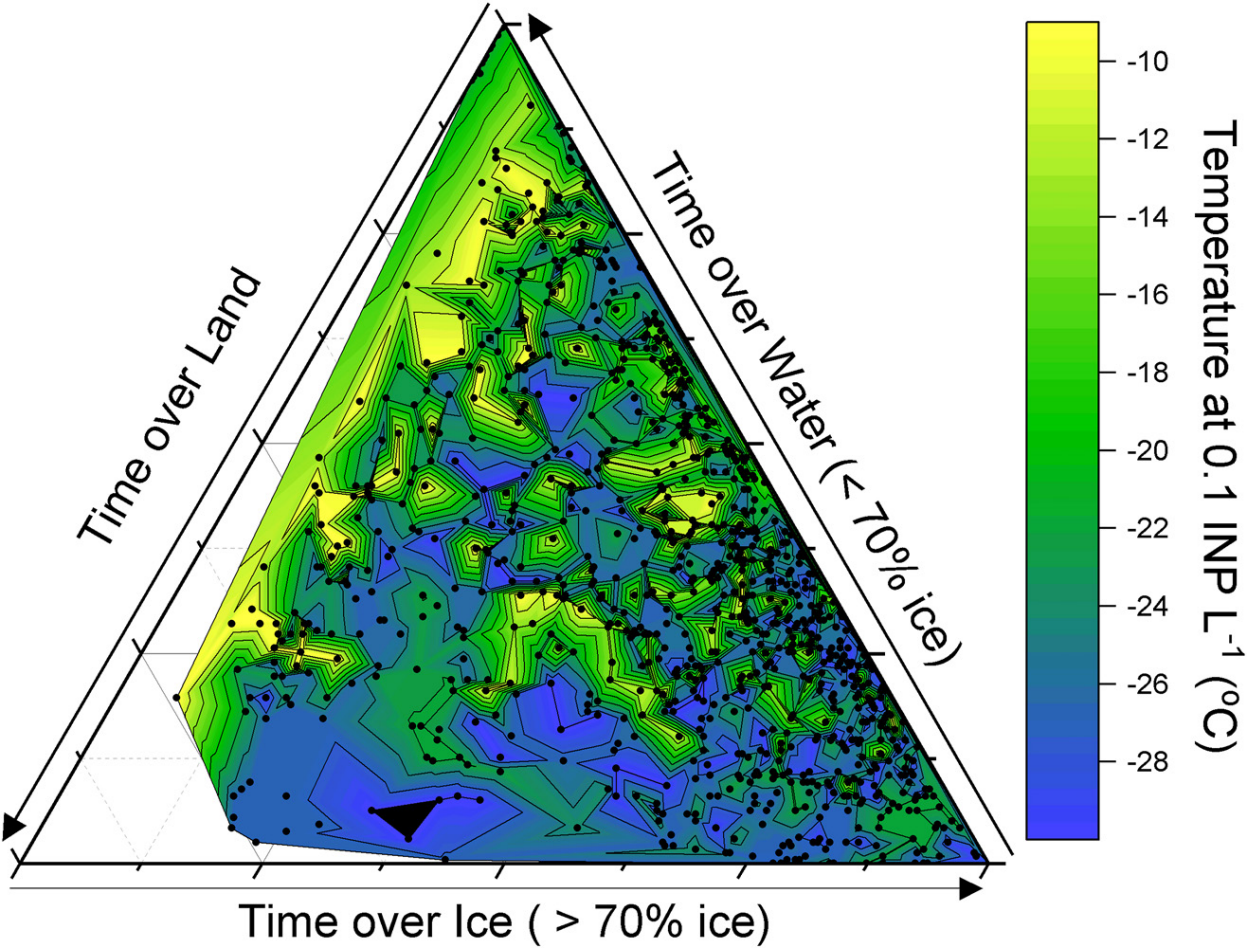
The time that air masses spent over water, ice and land derived using back trajectory analysis. The color represents the temperature at which an INP concentration of 0.1 INP L^−1^ was reached. The black dots correspond to individual trajectories. The same filters as in Figure 5 were applied.

Some of the back trajectories that had the highest INP concentrations passed over islands in the Barents and Kara Seas, including Svalbard, Franz Josef Land, Novaya Zemlya and Severnaya Zemlya. Many of these locations have been identified as poorly defined dust sources (Bullard et al., 2016; Meinander et al., 2021) and dust from Svalbard has been shown to contain biological ice‐nucleating materials (Tobo et al., 2019). However, in a further analysis of the back trajectory data (Figure 6), we find that there was little to no correlation with time spent over land, whereas the ice‐nucleating activity increased with the time the air parcels spent over open ocean. This implies that the sources of INP were associated with the marine environment. Having said this, we cannot rule out relatively small island point sources being important for INPs.

Overall, the evidence indicates that there is a strong source of biogenic INPs in the Barents, Kara and Laptev Seas off the Russian coast that can be sporadically transported to the central Arctic Ocean. There was high wind along the trajectories off the Russian coast which would be consistent with both the production of INPs in sea spray from these organic‐rich seas or dust combined with terrestrial biogenic material from the various islands in this region. We also note that a recent study found that very active INP were produced in the Chukchi sea (near Alaska) under high wave conditions (Inoue et al., 2021). Furthermore, we note that these potential sources are sensitive to a changing climate, with river discharge, permafrost melt and organic matter input into the ocean from the major Russian rivers increasing in a warmer world (Jahn et al., 2020). In addition, the removal of ice from the Arctic could also expose marine and terrestrial sources of ice‐nucleating material around the Arctic Ocean, where it can then be aerosolized by the action of wind (Schmale et al., 2021).

### 3.5 Implications for Ice Production in Boundary Layer Central Arctic Mixed‐Phase Clouds

In this section, we assess whether the measured INP concentrations are high enough to initiate a transition from liquid‐dominated clouds to ice‐dominated clouds. Model simulations indicate that on the order of one ice crystal per liter of air is required to remove the bulk of liquid water from an Arctic cloud, whereas lower concentrations still reduce the liquid water path (Stevens et al., 2018; Vergara‐Temprado, Miltenberger et al., 2018). Hence, we use our INP measurements, both at ship level in the surface mixed layer and those from the SHARK in the cloud mixed layer, to estimate the concentration of INPs that become active at the ambient temperature of the atmosphere ([INP]_ambient_).

The quantity [INP]_ambient_ combines the atmospheric temperature profiles from radiosonde measurements (six hourly) with the corresponding INP spectra as a rough indicator of primary ice crystal production. This is a crude analysis – a full cloud model would be required to represent ice crystal formation and sedimentation as well as INP recycling and latent heat release, and to predict ice crystal concentration given an INP spectrum – but it does give an indication of what the measured INP spectra might mean for primary ice production in clouds. The highest [INP]_ambient_ will be at the coldest points, that is, the top of the surface mixed layer and the top of the boundary layer (top of the cloud mixed layer), hence this is where we focus this analysis. We assume that ship level measurements are representative of the [INP]_ambient_ at the top of the surface mixed layer and those from SHARK are representative of the top of the boundary layer, the assumption being that these respective layers are individually well‐mixed.

**Figure 7:**
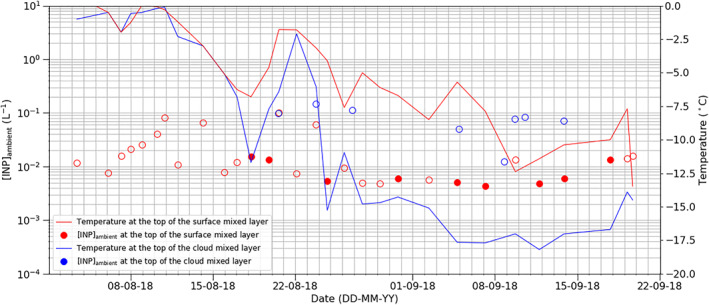
Time series showing [INP]_ambient_ for both the measurements taken at ship height (within the surface mixed layer) and using the SHARK (within the cloud mixed layer). The temperature at the top of the mixed layers is shown alongside these measurements. When an INP spectrum did not extend to the atmospheric temperature (i.e., where the highest temperature at which an INP concentration was reported was below the atmospheric temperature), the [INP] value associated with the highest temperature is provided as an upper limit to [INP]_ambient_ (open symbols).

The temperature minima within the main boundary layer were determined from routine radiosonde profiles with Väisälä RS92 radiosondes, which were made every six hours throughout the entire cruise (Vüllers et al., 2021). The calculated [INP]_ambient_ for the duration of the cruise are shown in Figure 7, where the minimum temperature at the top of the cloud mixed layer (main boundary layer) and the top of the surface mixed layer is indicated for each filter period. In many cases, the temperature of the atmosphere was higher than the temperatures at which we report the INP concentrations, hence we are only able to report upper limits to [INP]_ambient_ in these cases.

Generally, [INP]_ambient_ was typically below about 0.1 INP L^−^^1^ at the top of both the surface mixed layer and the top of the cloud mixed layer (and since many of the values in Figure 7 were upper limits, the [INP]_ambient_ might be much smaller than 0.1 L^−^^1^). The periods of high INP concentration before the 23rd August coincide with periods of higher ambient inversion temperatures, whereas later in the campaign the opposite is the case, which results in a relatively invariant [INP]_ambient_. Whether this is a coincidence, or if INP concentrations are correlated with ambient temperature, is unclear from this limited data set. However, there may be a physical mechanism behind this apparent correlation. Transport of air to the North Pole from sources further afield will likely result in multiple cycles of cloud formation and dissipation in any one air mass, hence INP active at above the ambient temperature of those clouds will likely activate and be removed via precipitation (bearing in mind that the majority of INPs relevant for mixed‐phase clouds only activate to ice in the presence of water droplets (Murray et al., 2012)). While transport through the boundary layer likely removes INP active above the lowest temperatures experienced by an air parcel, further cooling subsequent to the measurement time will lead to primary ice production. Hence, the air masses sampled before the 23rd August with high INP concentrations have a great potential for primary ice production if or when these air masses become colder downwind of the sampling location. In the absence of local sources, the INP spectrum over the central Arctic Ocean must therefore be determined by a combination of the characteristics of the upwind sources and the cloud temperature that these air parcels experience during transport.

The relatively low [INP]_ambient_ values suggest that clouds (assuming that they are not influenced by seeding from above) should be mixed‐phase, that is, contain a substantial proportion of liquid water with some ice crystals. Observations of the phase of clouds during this campaign are discussed in Vüllers et al. (2021). Overall, the fraction of single layer clouds (where seeding from above is unlikely) throughout the troposphere that were mixed‐phase was relatively constant throughout the campaign: mixed phase frequency ∼20%‐30% (of the campaign period), ice cloud frequency ∼10%‐30% and liquid clouds were generally infrequent (see Figure 15 in Vüllers et al. (2021)). The mixed‐phase frequency was relatively constant despite the strong decrease in temperature during the campaign. Similarly, in the bottom few kilometers the frequency of occurrence of mixed‐phase clouds were often between 25% and 50%, with ice clouds about half as frequent and liquid only clouds much less frequent throughout the campaign. Overall, the observations of a lack of clear trend in cloud phase for single‐layer clouds reported by Vüllers et al. (2021) are consistent with our relatively invariant [INP]_ambient_ measurements.

Clouds were multi‐layered around 50% of the time, with many situations identified where seeding of ice from higher, colder clouds into lower clouds might occur. Clouds were regularly observed up to around 8 or 9 km, where temperatures were low enough for homogeneous freezing (Herbert et al., 2015), and frequently occurred in the mid‐troposphere where heterogeneous nucleation on INPs was most likely important. These higher clouds were often in the free troposphere where our boundary layer INP measurements are not necessarily relevant. Indeed, aerosol and INPs in the free troposphere may have different sources to those in the boundary layer. In order to obtain a more complete picture of primary ice production in clouds in the central Arctic, INP measurements in the free troposphere in this region would be needed and should be a target of future campaigns.

## 4 Summary and Conclusions

Arctic mixed‐phase and supercooled clouds play a crucial role in Arctic climate, but the processes that dictate their characteristics are poorly understood. Here, we show that INP concentrations at 88–90°N are extremely variable, and throughout the MOCCHA campaign between the 1st of August 2018 and the 18th of September 2018 the temperature at which 0.1 INP L^−^^1^ was reached varied between −9°C and −30°C. The highest 20% of observed INP activity is related to air masses originating in the ice‐free ocean environment off the Russian coast, while the lowest 37% of observations related to air masses which originated and circled over the pack ice north of Canada for most of the 7‐day back trajectory. Trajectories of air with intermediate INP activity also originated over the ice‐free ocean. These results indicate a strong dependence of the measured INP concentration on the origin of the air with pack ice, open leads, and the MIZ apparently being weak sources of INP, whereas ice‐free oceans, especially those near the Russian coast when wind speeds were high, were a significant source.

The heat sensitivity of the most active INPs indicates the INP to be of biogenic proteinaceous origin. This, together with the trajectory analysis, indicates that there are strong biogenic sources of INP in the shallow seas over the Russian continental shelf. The ice‐nucleating activity of the aerosol at the North Pole derived from off the coast of Russia is much greater than that for sea spray aerosol from other oceans (including the Southern Ocean (McCluskey, Hill, et al., 2018) or the North Atlantic (McCluskey, Ovadnevaite, et al., 2018)). This may indicate the marine waters off Russia are very rich in ice‐nucleating material, perhaps related to the substantial riverine input, or alternatively the islands in this region may be sources of biogenic INPs. More work is needed to define what the key sources are along the Russian coast and to see if similar sources exist elsewhere around the Arctic and Antarctic.

By making measurements of INP spectra both above and within the surface mixed layer of decoupled boundary layers, we found that surface measurements were often not representative of the INPs in the cloud mixed layer. Hence, measurements at altitude, within the cloud mixed layer, are necessary in order to define primary ice production in Arctic mixed‐phase clouds. In addition, our measurements allowed us to estimate the INP concentration active at the temperature of the top of the surface mixed layer and also at the top of the boundary layer. This revealed that, despite massive variability in INP spectra, the INP concentration at ambient temperature was typically less than 0.1 L^−^^1^, which is consistent with remote sensing observations that indicate the persistence of mixed‐phase clouds (in the absence of seeding of ice from above). We also recommend future studies focus on INP measurements throughout the free troposphere where primary ice production may lead to seeding of ice in lower level clouds.

Overall, it is striking that INP concentrations at the summertime North Pole vary from some of the lowest measured anywhere in the world, to as high as the highest INP concentrations in terrestrial locations rich in biological INPs such as in the UK (O'Sullivan et al., 2018). Since these INPs are transported from the seas off the Russian coast, they may be sensitive to changes in climate. In particular, reduced sea ice, land ice, and permafrost may open up more INP sources for more of the year around the Arctic, which may increase the future strength (and may already have done so) of the sources of INPs that are important for mixed‐phase clouds in the central Arctic. More work needs to be undertaken to understand how climate change may affect INP sources around the periphery of the Arctic and how this may influence Arctic clouds and feedback on Arctic climate.

## Conflict of Interest

The authors declare no conflicts of interest relevant to this study.

## Supporting information

Supporting Information S1

## Data Availability

The ice‐nucleating particle data associated with this paper is available from University of Leeds data repository at (https://doi.org/10.5518/1093; Porter et al., 2022). The fine mode aerosol size distribution data is available from the Bolin database at https://doi.org/10.17043/oden-ao-2018-aerosol-dmps-1 (Karlsson & Zieger, 2020), the coarse mode aerosol size distribution data is available at https://doi.org/10.17043/oden-ao-2018-aerosol-coarse-1 (Zieger & Karlsson, 2022a) and the black carbon data is available at https://doi.org/10.17043/oden-ao-2018-aerosol-coarse-1 (Zieger & Karlsson, 2022b). In addition, there is an overview of all available data from the Arctic Ocean expedition, 2018 (Leck et al., 2018).
