# Supplementary material for: Highly Active Ice‐Nucleating Particles at the Summer North Pole
Source: J Geophys Res Atmos. 2022 Mar 17;127(6):e2021JD036059. doi: 10.1029/2021JD036059 (PMC9285974; doi:10.1029/2021JD036059)
Supplement: Supplementary file 1 — Supporting Information S1 [file JGRD-127-0-s001.docx]

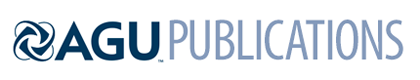


*Journal of Geophysical Research: Atmospheres*

Supporting Information for

**Highly active ice-nucleating particles at the summer North Pole**

Grace C. E. Porter^1,2^, Michael P. Adams^1^, Ian M. Brooks^1^, Luisa Ickes^3^, Linn Karlsson^4,5^, Caroline Leck^4,6^, Matthew E. Salter^4,5^, Julia Schmale^7^, Karolina Siegel^5,6,4^, Sebastien N. F. Sikora^1^, Mark D. Tarn^1,2^, Jutta Vüllers^1^, Heini Wernli^8^, Paul Zieger^4,5^, Julika Zinke^4,5^ and Benjamin J. Murray^1^

^1^ School of Earth and Environment, University of Leeds, Leeds, UK

^2^ School of Physics and Astronomy, University of Leeds, Leeds, UK

^3^ Department of Space, Earth and Environment, Chalmers, Gothenburg, Sweden

^4^ Bolin Centre for Climate Research, Stockholm University, Stockholm, Sweden

^5^ Department of Environmental Science, Stockholm University, Stockholm, Sweden

^6^ Department of Meteorology, Stockholm University, Stockholm, Sweden

^7^ School of Architecture, Civil and Environmental Engineering, École Polytechnique Fédérale de Lausanne, Lausanne, Switzerland

^8^ Institute for Atmospheric and Climate Science, ETH Zürich, Zürich, Switzerland

**Contents of this file**

Figs. S1 to S3

Tables S1 to S3

**Introduction**

- This document contains some plots and tables that support the main paper.


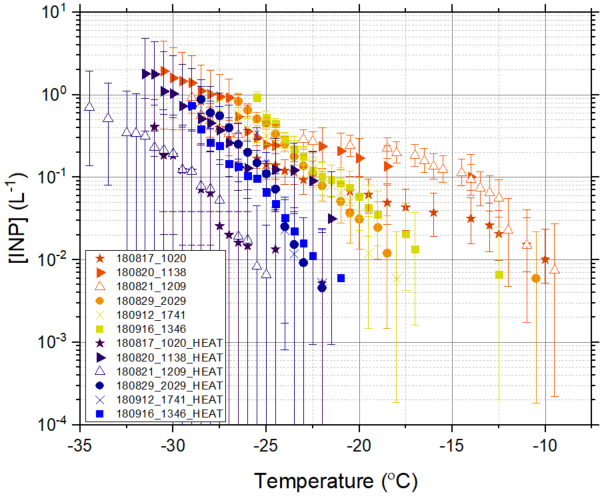


**Figure S1.** INP concentration results for heat-tested samples throughout the campaign. The spectra shown in blues are for samples that were immersed in a boiling water bath for 30 min, whereas the spectra for the corresponding unheated samples are shown in yellows and oranges (from Figure 1). The data have had the backgrounds subtracted. Temperature uncertainties (not shown) for the droplet freezing experiments were estimated to be ±0.4 °C. The legend is defined as YYMMDD_time, representing the starting time of the sampling period in the 24-hour format.


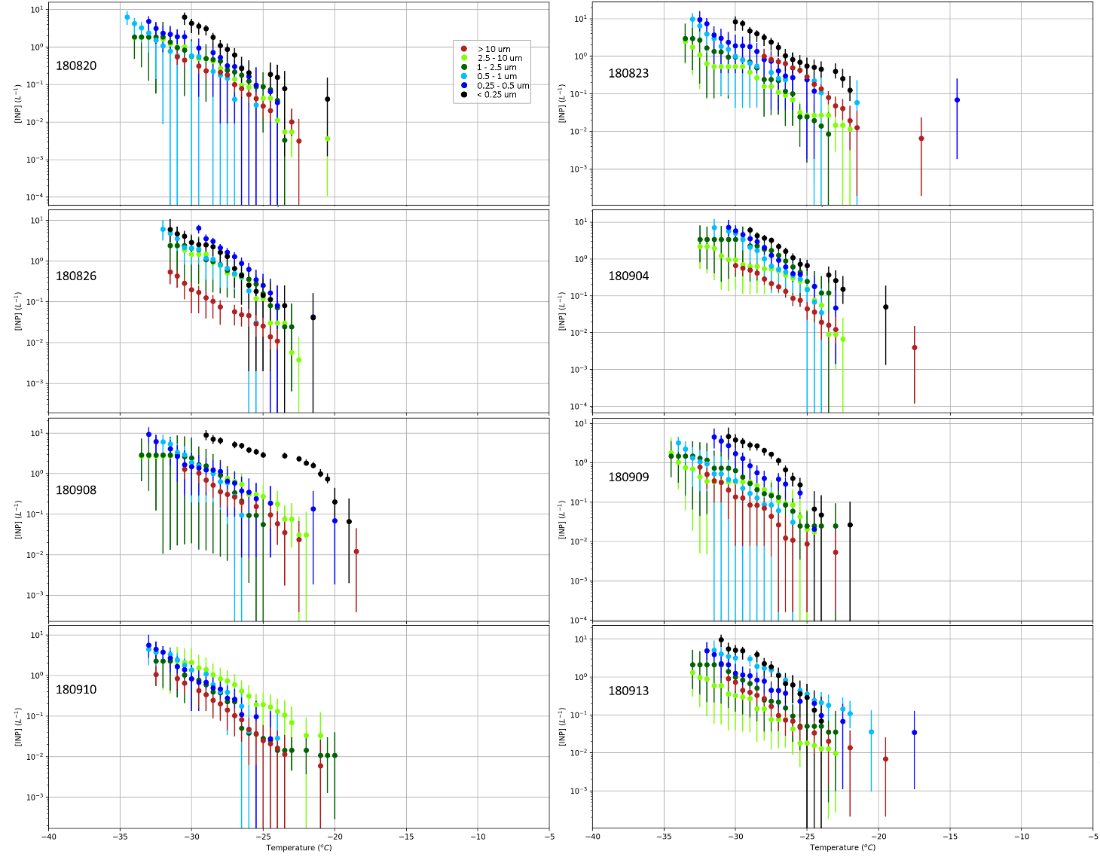


**Figure S2.** Size-resolved INP spectra from each SHARK flight in the cloud mixed layer. These data are background subtracted. The two cascade impactors on the SHARK had stages with varying aerosol size cut-offs/bins. The two size ranges which overlapped for both impactors were 1-2.5 μm and 2.5-10 μm. These size ranges were combined by taking the average of the INP concentrations of each impactor in each temperature bin.


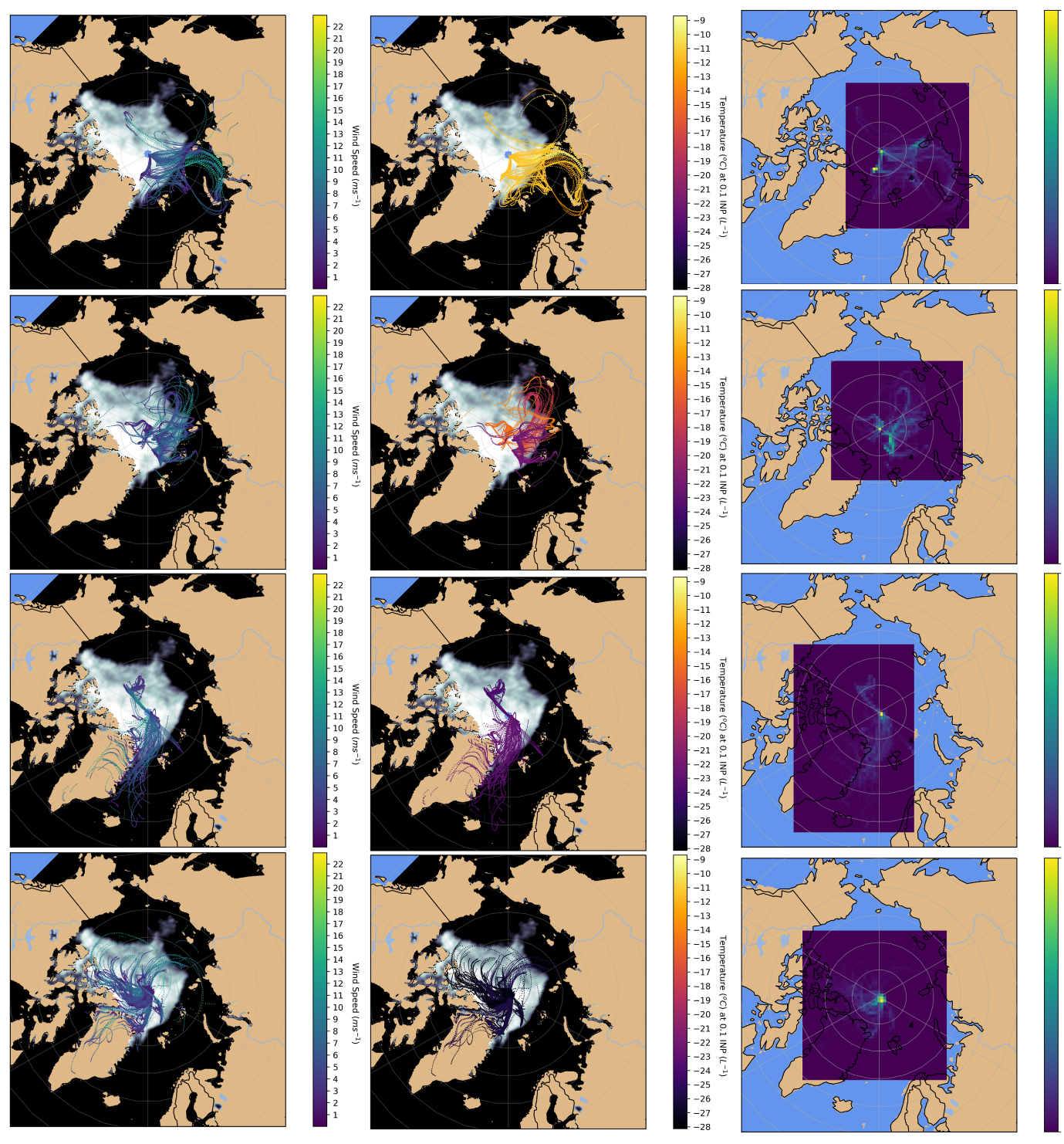


**Figure S3.** Backward trajectories over 7 days with the colour indicating wind speed (left column) and temperature at 0.1 INP L-1 (right column). Each row is for a subset of the INP measurements, with the samples with the most active INP in the top pair, and the lowest in the bottom pair.

| Start time | End time | Sampling time / hrs |
| --- | --- | --- |
| 02/08/2018 23:00 | 03/08/2018 21:15 | 22.25 |
| 05/08/2018 11:50 | 06/08/2018 16:55 | 29.08 |
| 06/08/2018 17:10 | 07/08/2018 15:55 | 22.75 |
| 07/08/2018 16:02 | 08/08/2018 08:30 | 16.47 |
| 08/08/2018 10:51 | 09/08/2018 11:10 | 24.32 |
| 09/08/2018 17:15 | 10/08/2018 18:28 | 25.22 |
| 10/08/2018 18:28 | 11/08/2018 00:20 | 5.87 |
| 11/08/2018 13:42 | 12/08/2018 10:46 | 21.07 |
| 13/08/2018 18:18 | 14/08/2018 13:24 | 19.10 |
| 15/08/2018 13:25 | 16/08/2018 09:30 | 20.08 |
| 16/08/2018 16:06 | 17/08/2018 10:19 | 18.22 |
| 17/08/2018 10:20 | 19/08/2018 01:02 | 38.70 |
| 19/08/2018 01:04 | 20/08/2018 11:29 | 34.42 |
| 20/08/2018 11:38 | 20/08/2018 17:50 | 6.20 |
| 21/08/2018 12:09 | 22/08/2018 17:34 | 29.42 |
| 23/08/2018 17:07 | 23/08/2018 20:30 | 3.38 |
| 23/08/2018 23:15 | 25/08/2018 11:41 | 36.43 |
| 25/08/2018 12:15 | 26/08/2018 20:00 | 31.75 |
| 26/08/2018 22:42 | 28/08/2018 11:40 | 36.97 |
| 28/08/2018 13:25 | 29/08/2018 20:29 | 31.07 |
| 29/08/2018 20:29 | 31/08/2018 15:12 | 42.72 |
| 31/08/2018 21:20 | 03/09/2018 20:20 | 71.00 |
| 03/09/2018 20:20 | 05/09/2018 17:15 | 44.92 |
| 05/09/2018 17:15 | 08/09/2018 14:57 | 69.70 |
| 08/09/2018 15:40 | 10/09/2018 19:24 | 51.73 |
| 10/09/2018 19:13 | 12/09/2018 17:40 | 46.45 |
| 12/09/2018 17:41 | 15/09/2018 00:15 | 54.57 |
| 16/09/2018 13:46 | 19/09/2018 00:52 | 59.10 |
| 19/09/2018 00:53 | 19/09/2018 11:00 | 10.12 |
| 19/09/2018 11:00 | 19/09/2018 23:15 | 12.25 |

**Table S1.** Date and time of the start and end of the ship-based filter samples on the whole air inlet.

| Date | Sampling period | SHARK measurement height (m) | Decoupling height  radiosonde (m) | Decoupling heights radiometer (m)  (hourly medians) |
| --- | --- | --- | --- | --- |
| 180820 | 1040-1530 | 400 | 100 (12UTC) | 85, 85, 85, 85, 85 |
| 180823 | 1720-2020 | 550 | 85 (18UTC) | 85, 85, 85 |
| 180826 | 1730-2220 | 450 | 300 (18UTC) | 320, 320, 450, 450, 450 |
| 180904-180905 | 2000-0015 | 390 | 470 (24UTC) | 320, 320, 350, 490 |
| 180908 | 1705-2005 | 500 | 440 (18UTC) | 290, 290, 320 |
| 180909 | 1315-1955 | 410 | 60 (18UTC) | 35, 35, 35, 35, 35, 35 |
| 180910 | 1015-1535 | 550 | 0 (12UTC) | 20, 20, 20, 20 |
| 180913 | 1745-2335 | 600 | 420 (18UTC) | 170, 930, 890, 890, 35, 85 |

**Table S2**. Date and time of SHARK sampling periods and estimated decoupling heights of the surface mixed layer from radiosonde (the six hourly radiosonde launches) and radiometer measurements (Vüllers et.al, 2021).


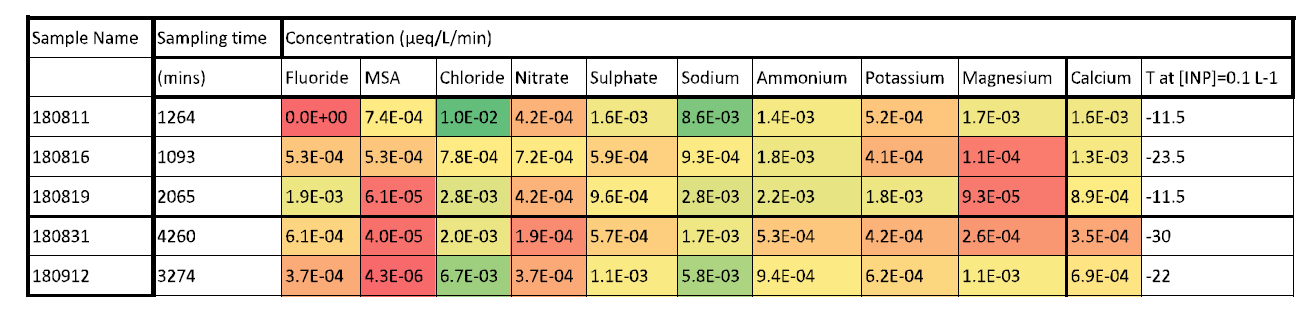


**Table S3**. Concentration of major water-soluble ions derived using ion chromatography for several of the filter samples. The aqueous samples were the same as those used for INP analysis where the aerosol was washed off the filters into a volume of water. The quoted concentration is the concentration of ions in water normalised to the sampling time.
